# Design and analysis of solitary AC–AC converter using reduced components for efficient power generation system

**DOI:** 10.1038/s41598-024-60230-3

**Published:** 2024-04-23

**Authors:** K. Nandakumar, V. Mohan, Faisal Alsaif, S. Senthilkumar

**Affiliations:** 1 Department of Electrical and Electronics Engineering, E.G.S. Pillay Engineering College, Nagapattinam, Tamilnadu 611002 India; 2 Department of Electrical Engineering, College of Engineering, King Saud University, 11421 Riyadh, Saudi Arabia; 3 Department of Electronics and Communication Engineering, E.G.S. Pillay Engineering College, Nagapattinam, Tamilnadu 611002 India

**Keywords:** AC–AC converter, AC grids, Power system, Buck-boost converter, Engineering, Electrical and electronic engineering

## Abstract

Considering different applications that require varied power and voltage conversion levels between AC grids and AC loads, AC–AC power conversion between AC grids has become an inevitable technology of energy management systems. An isolated converter for performing AC-to-AC transmission is proposed with minimal components for reduced losses and enhanced system efficiency. Single-phase direct buck-boost AC to AC converter with minimum components constituted with two dual IGBT control units (IGBT 1–IGBT 4), inductor (L_f_), and capacitor (C_f_) is proposed in this work. The MATLAB/Simulink platform is used to provide in-depth analysis of the circuit and components along with the design guidelines, and simulation outcomes of this proposed model. The voltage gains of G = 2.13, power factor of 0.97, and overall efficiency of 98% are achieved in the proposed system with minimum components of 4 switches, 2 conductors, and 1 capacitor and inductor respectively. The obtained results are compared with existing technology to evaluate the proposed system.

## Introduction

AC–AC converters play an important role in industry since they are often used in machine speed control, along with low frequency and variable voltage magnitude. Due to their significance, it has become a current research topic in the evolution of AC-to-AC converters. Commonly, this type of conversion is accomplished using thyristor-based controllers to generate the correct output voltage by refining phase angle^1–5^. An AC waveform can be converted to another AC waveform with arbitrary output voltage and frequency settings using a solid-state AC–AC converter. It is possible to realize an AC–AC converter with bidirectional power flow and input currents that are roughly sinusoidal by connecting a PWM rectifier and an inverter to the DC link.

**Figure 1:**
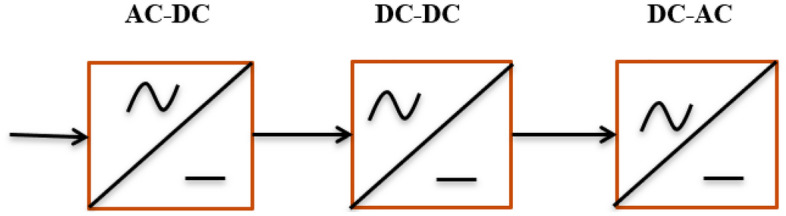
Basic AC–AC converter with 3 stages.

The energy storage component shared by both stages, which is either an inductor L for the current DC-link or a capacitor C for the voltage DC-link, then impresses the DC-link quantity. Moreover, the PWM inverter stage has a consistent, AC line-independent input quantity, which leads to a high utilization of the converter's power capacity (Szcześniak et al. 2015). However, the DC-link energy storage element has a comparatively high physical volume, and in the case of a voltage DC-link, there may be a shorter system lifetime if electrolytic capacitors are utilized. Figure 1 shows the types of three-phase AC–AC converters. Figure 1 shows the basic AC–AC conversion steps.

However, this type of circuit has numerous problems like system efficiency and harmonic disturbances leading to the establishment of filters in the system. Pulse Width Modulators (PWM) based converters discussed in^6–11^ alleviate the disadvantages of thyristor-based AC controllers by providing a means to tackle commutation complications and offering more efficiency. A pulse width modulation technique for conversion was adopted by^6^, and commutation complications are handled by utilizing the changing cell topology and combined inductors. Further, it is performed by utilizing the Z impedance, which allows protected commutation and a larger value of output voltage. Furthermore, the converters proposed by^8–14^ were the revised ones than the devices given by^6,7^ which are supposed to be free of issues. In^8^, they use magnetic integration to remove the filter from changing cell AC to AC converters. To achieve high voltage levels, several different changing cell methodologies were proposed^9^. In addition, Kim et al.^12^ introduces the usage of changing multilevel AC to AC converter to restore potential differences.

PWM-based AC to AC converters attracted researchers in recent years, because of their output elevated efficiency, Simplified device structure, improved power factor, lower noise, easy device control, and reduced i/p and o/p filter setup when compared to thyristor-based controllers. Among the commonly used three types of PWM AC to AC converters, the resulting voltage along with the frequency of both in-direct and matrix-oriented converters can be adjusted^15–18^. Matrix-oriented converters necessitate greater units of semiconductor control, resulting in low efficiency as well as large size and expense. Furthermore, it has severe commutation issues as discussed in^19–22^. Whereas the direct type converter is based on one-step power conversion which can vary the required voltage. Since the conversion is done in one stage, with reduced size and economic nature, this type is used for regulating the resulting voltage^23^. Common direct mode converters evolved through common DC converters by substituting bidirectional control for unidirectional control^24–26^. Because of the flap time duration with the end time duration that happens over complimentary control units, all of these topologies have commutation issues. Current and voltage spikes are caused by the flap time duration with end time duration, which destroys semiconductor controls. In^3^, a series of traditional Z-source-based AC to-AC converters that may step up the fed-in voltage demand and a complicated control technique to solve the commutation concerns were discussed. To address the limitations of the general Z-source AC to AC converter which is studied and analyzed in^2–5^ the modified ostensible Z-source AC to AC converter was developed.

**Figure 2:**
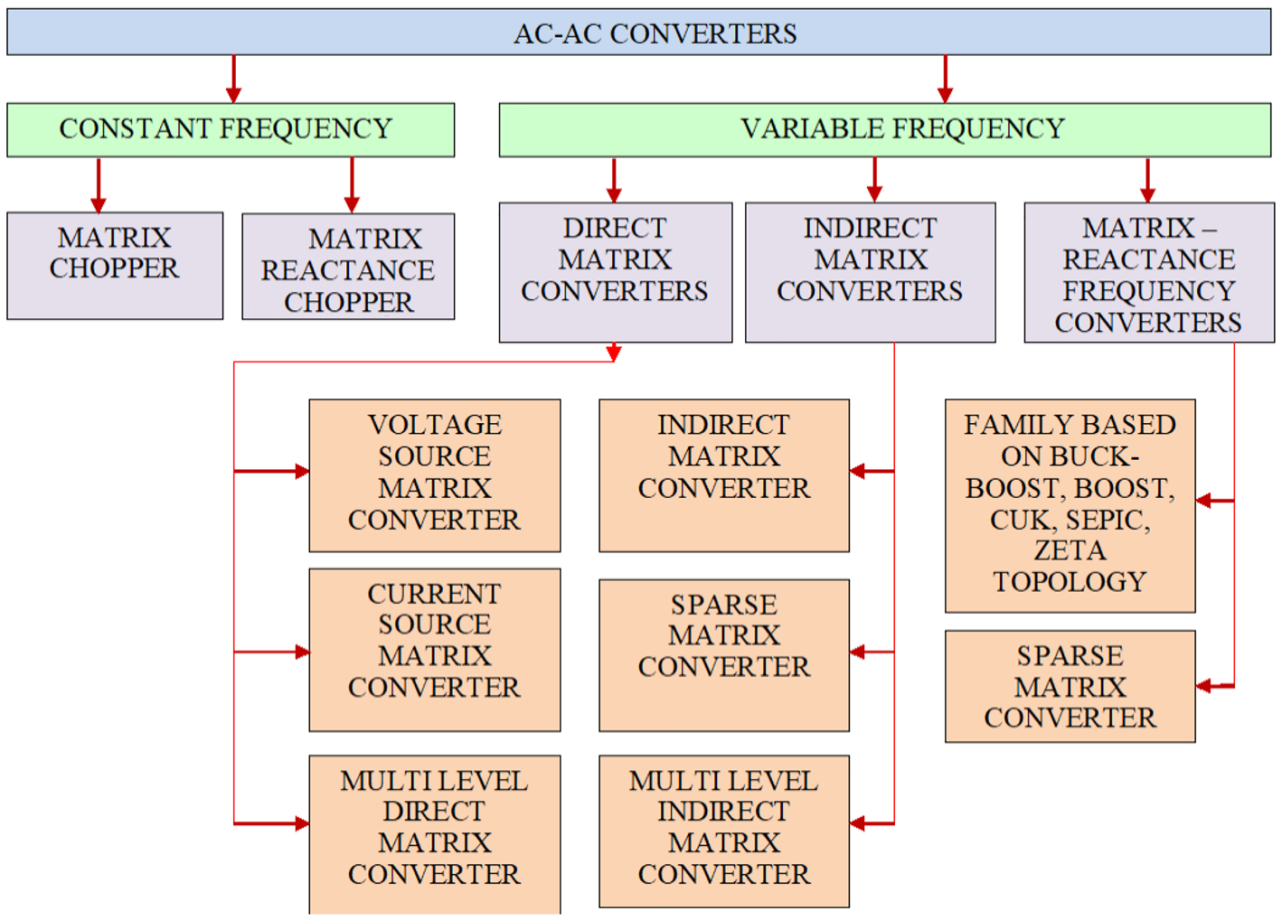
Types of three phase AC–AC Converters.

Single-phase converters were built in^27^ by substituting the standard PWM converter's bidirectional switches with the linked inductor and the control cell topology. Even though the above-mentioned type eliminates the reverse recovery issues and solves the commutation problem, these mentioned type converters are affected by circulating current components resulting in greater losses, and strain control, which degrades the overall efficiency of the system. As discussed by^28^, a converter set is designed to overcome to compensate for the imperfection of the type of converters discussed in^27^. Rotating the magnetic field with the current of connected inductors is minimized by substituting the inductors present in the circuit using a very small inductor. Later^29^ introduces a 1Φ buck-boost AC to AC conversion by using reduced components. This modified system has a sophisticated swapping technique that necessitates the use of dual capacitor units during dead time and to overcome difficulties. A novel topology of Z-source AC to AC converter was discussed in^30–32^ by substituting the components which were proposed by^29^. This device is made up of four control units and diodes respectively which necessitate the use of dual capacitors, each connected parallel to minimize the inference over input voltage in the circuit. The discussed converters essentially need a high number of active, passive switches, which escalates the area and expense of the converter with the decrease in its overall performance. Figure 2 represents the different types of AC–AC converter.

Ahmed et al. ^33^ Introduces a 1Φ AC to AC converter to correct the grid potential, which requires a large quantity of components. This topology employs six control units, and diodes, supported with each inductor, and capacitor. As described by^34–36^, the converter of direct mode can further be used for potential compensation and output power regulation. Many studies are being conducted to improve the tame and adaptability of the converters. In^36^, for straight AC to AC power conversion, a spurious 2Φ input voltage-based AC voltage generation way with supporting regulation methodology is described^37–39^. A 1Φ AC to AC converter with minimum components works as a converter is introduced in this research. Every method of operation uses only one control function with the diode of another control unit, reducing circuit losses. The suggested converter has no commutation issues because the input value of the current is constant. Furthermore, even if the complete switches are turned on at the same moment, there is no chance of the input source shooting through.

## Proposed AC to AC converter

**Figure 3:**
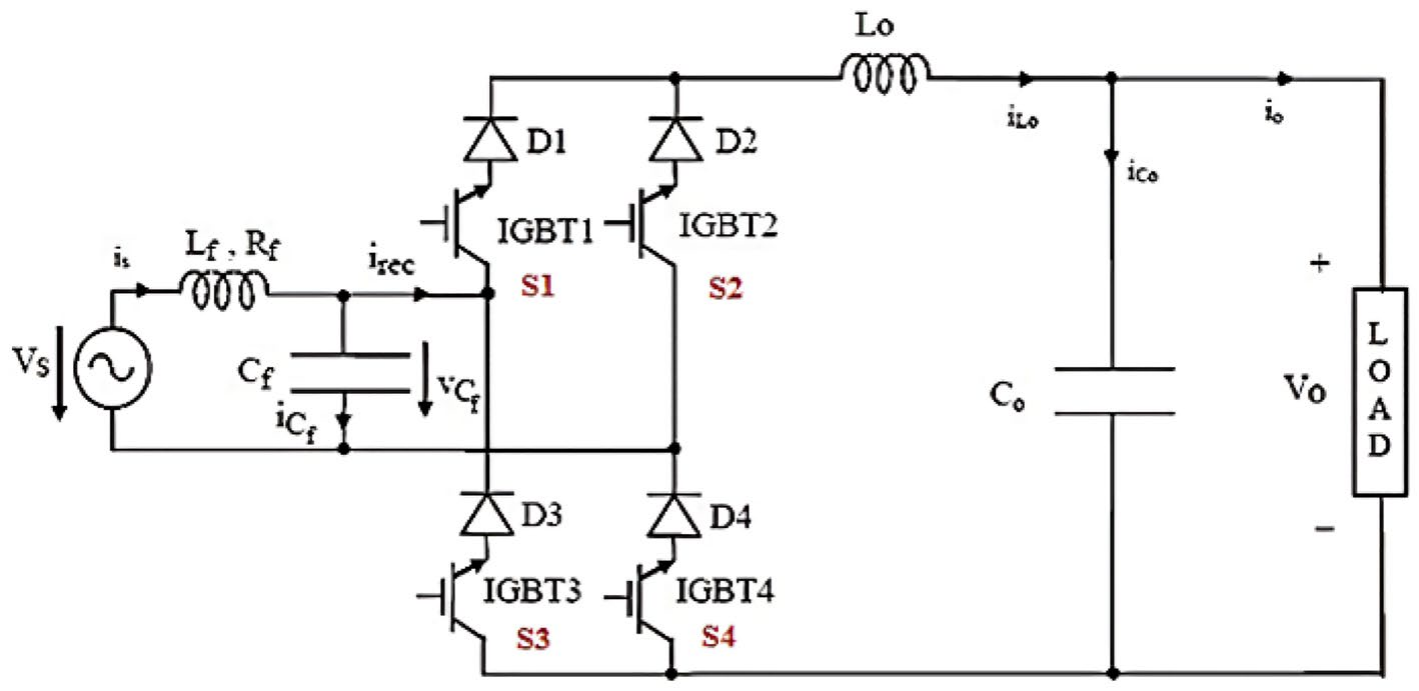
1-Φ direct buck-boost AC to AC converter.

Figure 3 represents the converter model discussed in this study with minimum components constituted with two dual IGBT control units (IGBT 1–IGBT 4), inductor (L_f_), and capacitor (C_f_). Lo and Co represent the input and output inductor and capacitor used for filter purposes respectively.

### Method of operation

**Figure 4:**
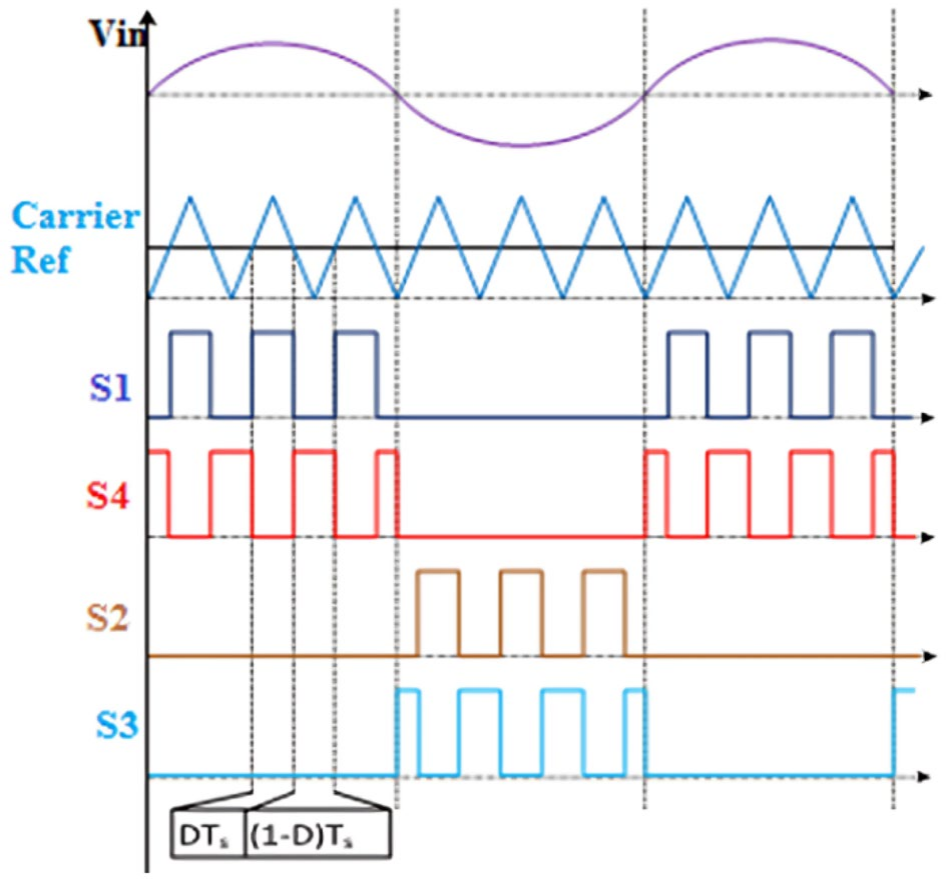
The waveform of trigger signals.

As given in Fig. 4, trigger signals are produced using a traditional carrier-based PWM technique. Where Ts gives the switch-over period and D represents the duty cycle. As depicted by Fig. 2, the modulated signal is applied to S1 and S2, while its equivalent is applied across switches S3 and S4. Every time the input voltage cycles in half, there are two operating modes. Only one switch is active at any given time, and in each mode of operation, the diode of a different control unit is biased (forward). The resultant high current representing a continuous waveform is noted.

### Various operating approaches

#### During positive half-cycle

##### Operating mode 1: [0-DTs]

**Figure 5:**
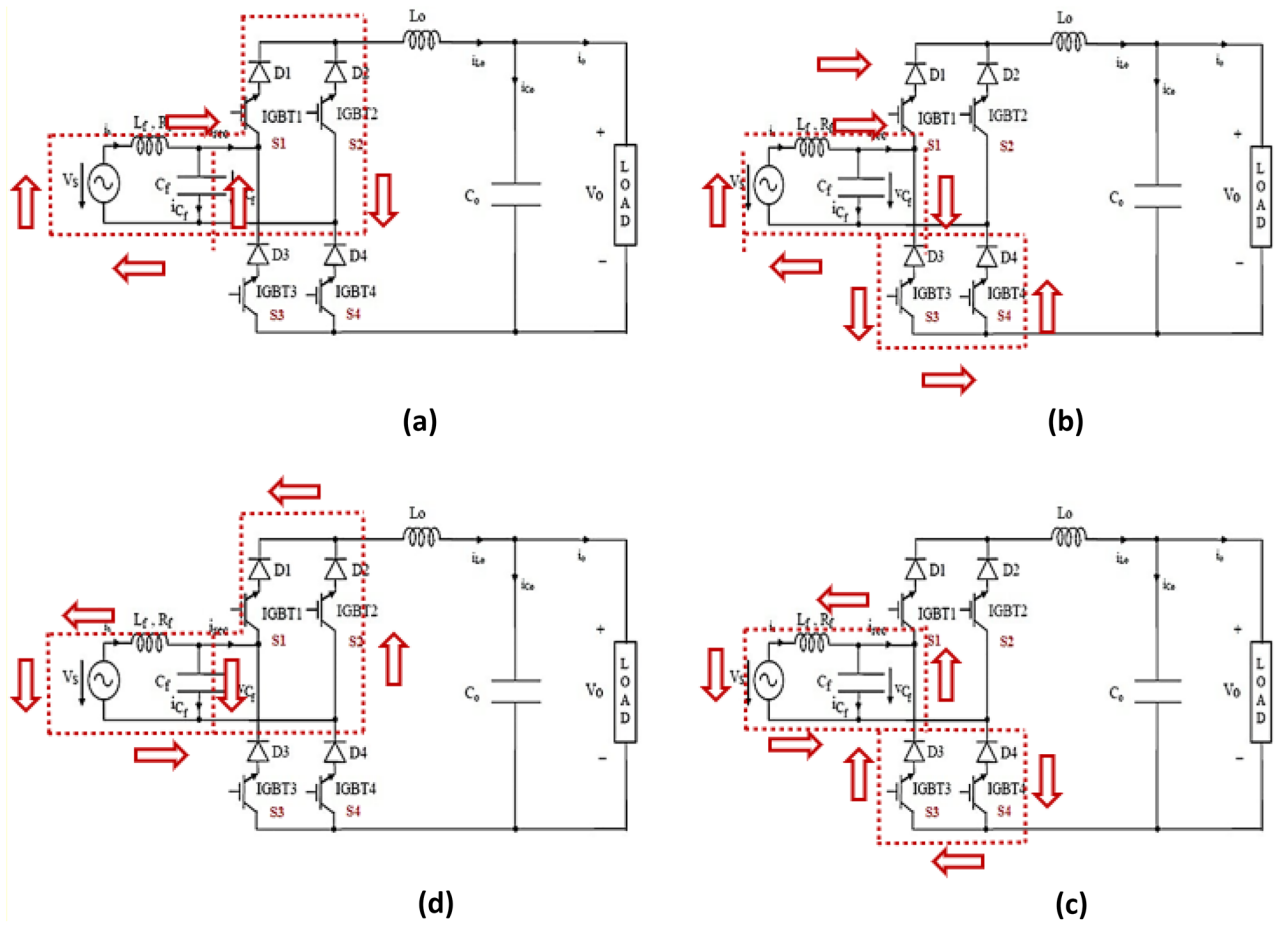
Representation of different operating modes.

As illustrated in Fig. 5a, switch S1 is turned on throughout the DTs interval. This created a conduit towards capacitor C1's stored energy to be discharged via L1. The input source is stored in inductor L1 and capacitor C1. When we apply KVL in steady-state operation to the circuit depicted in Fig. 5a, we get

1 $$vL1=vC1$$

2 $$vC1=vin$$

where v_in_ is the voltage input and L_in_ gives the potential drop.

##### Operating mode 2: [DTs-Ts]

The control unit S4 is powered on during this type of operation, and the diode will be forward biased for (1-D) Ts time interval, given in Fig. 5b. The energy of L1 is given to load as the source further recharges the capacitor C1. When we apply Kirchoff’s Voltage Law, we get

3 $$vL1=-{v}_{0}$$

4 $$vC1=vin$$

The voltage gain (G) can be formulated using the above equations as,

5 $$G=\frac{{v}_{0}}{{v}_{in}}=\frac{D}{1-D}$$

#### During negative half-cycle

The reported converter operates the way same as the + ve half-cycle converter. Figure 5c illustrates, the inductor L1 stores the energy from the source where capacitor C1 establishes a way to the body diode of S1 by S2. Figure 5d shows how the energy discharges through S3 into the load.

### Ethical approval

This paper does not contain any studies with human participants or animals performed by any of the authors.

## The proposed converter's parameter design

The maximum tolerated passive components ripples are primarily designed, and ripples of current and voltage are calculated using Eq. (6),

6 $${v}_{r}=L\frac{\partial {i}_{l}}{\partial t}$$

7 $${i}_{r}=C\frac{\partial {v}_{c}}{\partial t}$$

The maximum acceptable current ripple of an inductor is calculated as a percentage of its RMS value of maximum current.

8 $${\partial I}_{L-max}=\alpha {I}_{l-rms}^{max}$$

To obtain the maximum current across inductor the Eqs. (1) and (8) is used in Eq. 6, the resultant inductor equation is given as,

9 $${L}_{1}={V}_{in-rms}\frac{D{T}_{s}(1-D)}{\propto {I}_{0-rms}}$$

10 $${L}_{1}={D}^{2}\frac{{V}_{in-rms}^{2}}{\propto {f}_{sw}{P}_{0}}$$

The overall permissible voltage ripple of capacitor C_1_ is defined using the percentage of the peak voltages across it. Substituting an interval (1-D)*Ts by Eqs. (4) in (7).

11 $${C}_{1}=\frac{{I}_{in}(1-D)}{{f}_{sw} \beta {V}_{in-rms}}$$

12 $${C}_{1}=\frac{(1-D){P}_{0}}{ \beta {f}_{sw}{{V}_{in-rms}^{2}}}$$

here V_in_ represents the input voltage, V_o_ represents the output voltage, D represents the duty ratio, Io_-rms_ gives rms value of the load currents, Po, f_sw_ is output power, the switching frequency respectively. The Maximum voltages and currents of the control units can be further estimated using Eqs. (13) and (14) to identify the requisite ratings of the proposed converter's semiconductor switches.

13 $${V}_{s1-s4\left(pk\right)=\sqrt{2}({V}_{in-rms}+{V}_{0-rms})}$$

14 $${I}_{s1-s4\left(pk\right)=\sqrt{2}\left({I}_{in-rms}+{I}_{0-rms}\right)}$$

### Power loss and efficiency calculations

#### Losses due to conduction

The conduction losses arising within an imperceptible period in the IGBT (Pcond) are calculated as the product of the switch's ON state voltage by the amount of current.

15 $${P}_{cond}=[{V}_{CEO}+{R}_{C}.i\left(s\right)] i(s)=[{V}_{CEO}i(s)+{R}_{C}.i{.\left(s\right)}^{2}]$$

where V_CE0_ gives the voltage value of the collector-emitter junction for nil current when the IGBT is turned on, R_C_ is the resistance at the collector–emitter junction when the IGBT is turned on, where i(s) represents the value of the current. Because of the mono functioning of IGBT, the overall losses that occur in the four IGBTs due to conduction are similar to the losses of mono IGBT which is operating constantly during the complete cycle. Because both the half cycles are comparable, the integration over a half of the periodic duration can give the average conduction losses which are given as:

16 $${P}_{cond}=\frac{1}{\pi }{\int }_{0}^{\pi }\left[{V}_{CEO}i\left(s\right)+{R}_{C}.i{\left(s\right)}^{2}\right]d\left(ws\right)=[{V}_{CEO}.{I}_{avg}+{R}_{C}.{I}_{rms}^{2}]$$

Here I_avg_ and I_rms_ represent the switch current's average and RMS values. Similarly, the diode's average conduction losses are given as:

17 $${P}_{cond. Davg}={V}_{D0}.{I}_{avg}+{R}_{D}.{I}_{rms}^{2}$$

The overall losses which are due to conduction are calculated using Eq. (18),

18 $${P}_{cond. total={P}_{cond.IGBTavg}+ {P}_{cond. Davg}}$$

### Losses in switching

The switch's switching losses (Psw) are stated as:

19 $${P}_{SW}=\left({W}_{on}+{W}_{off}\right).{f}_{sw}$$

here Won and W_off_ denote energy dissipated during operation as discussed in ^37^. The suggested converter has dual switches that work separately during each half cycle, with the overall switching losses throughout the entire cycle.

20 $${P}_{SW.avg}=2.({P}_{SW})$$

### Calculation of power losses in passive components

Equations (21), (22), and (23) give the losses in (L_in_), (L1), and (C1) respectively,

21 $${P}_{L.in}={R}_{L.in}.{I}_{in-rms}^{2}$$

22 $${P}_{L1}={R}_{L1}.{I}_{L1-rms}^{2}$$

23 $${P}_{C1}={R}_{C1}.{I}_{C1-rms}^{2}$$

where R_L.in_, R_L1_, and R_C1_ denote the resistances of the inductor (L_in_), inductor (L_1_), and capacitor (C_1_). The overall passive power losses are calculated as shown in Eq. (24),

24 $${P}_{passive.losses}={P}_{L.in}+{P}_{L1}+{P}_{C1}$$

### Efficiency estimation

The power of the system is stated as follows:

25 $${P}_{0}={R}_{0}.{I}_{0-rms}^{2}$$

The total losses considering components and switches can be calculated as,

26 $$ {\text{P}}_{{{\text{cond}}.{\text{total}}}} + {\text{P}}_{{{\text{sw}}.{\text{avg}}}} + {\text{P}}_{{{\text{passive}}.{\text{losses}}}} = {\text{P}}_{{\text{Converter losses}}} $$

Equation (27) gives the input power of the converter,

27 $$ {\text{P}}_{{\text{o}}} + {\text{P}}_{{{\text{Converter}}\;{\text{losses}}}} = {\text{P}}_{{{\text{in}}}} $$

Equations (25) and (27) can be used to compute the converter's efficiency as follows:

28 $$Efficiency\%=\frac{{P}_{0}}{{P}_{in}}\times 100$$

### Power factor calculation

The power factor can be calculated by the equation given,

29 $$PF=\frac{{P}_{in}}{{V}_{in-rms}-{I}_{in-rms}}$$

## Results and discussion

Here for the study MATLAB software and the Simulink platform were adopted for analyzing the performance and efficiency of the converter. Table 1 summarizes converter parameters with a frequency of 3 kHz.

**Table 1:** Parameters of the converter operating at *f*_*sw*_ = 3 kHz.

Sl. no	Components	Range
1	Capacitor (*C*_1_)	14 µF
2	Inductor (*L*_1_)	9.2 mH
3	I/P inductor (*L*_*in*_)	8 mH
4	O/P capacitor (*C*_*out*_)	20 µF
5	I/P voltage (*V*_*in*_)	100 V_rms_/50 Hz
6	Resistance load (*R*_*o*_)	100 Ω
7	Inductance load (*R*_*o*_ and *L*_*o*_)	100 Ω & 150 mH

### Resistive load simulation results

**Figure 6:**
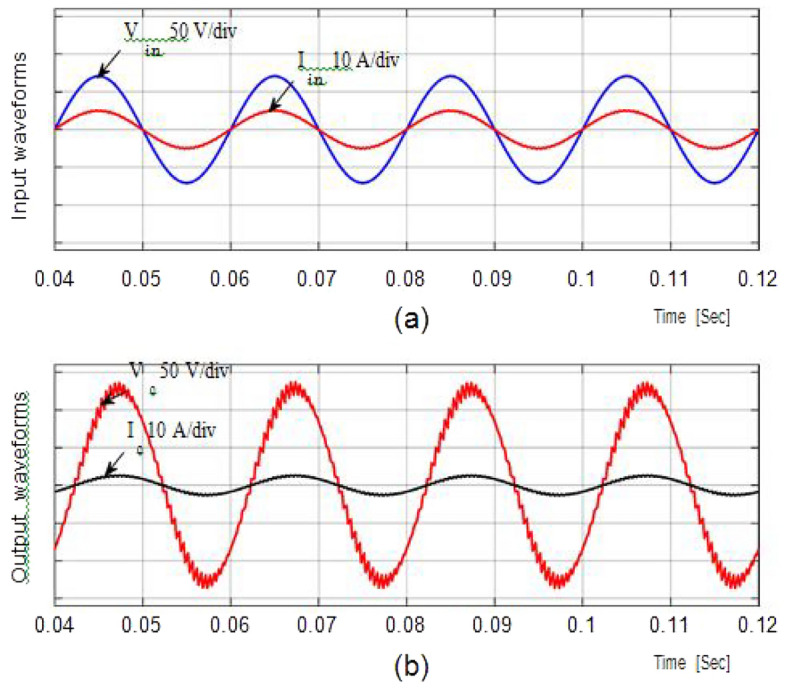
Output current and voltage.

From Fig. 6, a 50 V alternating current supply is fed into the system and is linked to a resistance load (Ro = 100Ω). The duty ratio is adjusted to 0.75 for boosting mode, and the corresponding waveforms are displayed in Fig. 4. With a voltage gain of 1.857, the output is around 96.32 V at an input voltage of 100. Voltage and current waveforms are also continuous.

Figure 5 depicts (iL1), (vC1), across S1, S2 at D = 0.75. The overall potential across control switches S1 and S2 is approximately 210 V. Figure 6 gives the Total Harmonic Distortion (THD) with an output voltage of 4.87% and 2.1% lies within the normal range.

**Figure 7:**
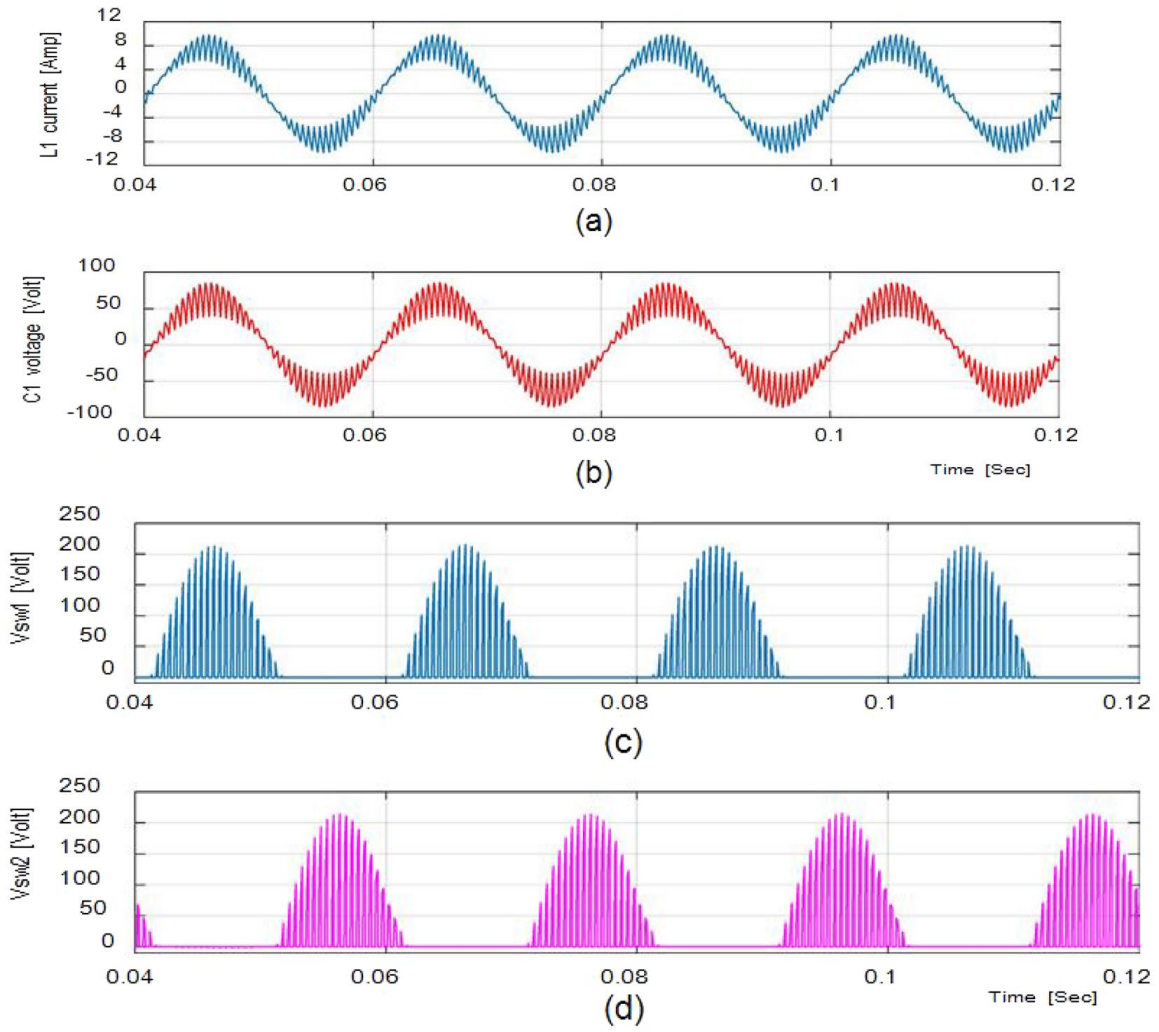
Simulation results (**a**) (i_L1_). (**b**) (v_C1_). (**c**,**d**) Potential across Switches.

**Figure 8:**
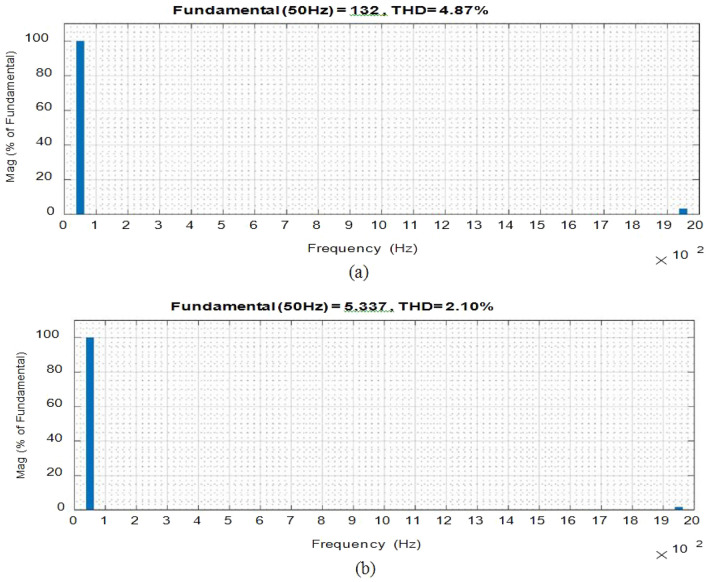
THD values (**a**) Output voltage. (**b**) Input current.

Figure 7a and b shows the result of the input and output voltages and currents in bucking mode with a ratio of 0.5 and an input voltage of 100 V. Because the output voltage is 18.76 V, the potential gain is 0.29. Figure 8a and b shows the inductor current (iL1), capacitor voltage (vC1), and voltage across S1, S2 at D = 0.5, with the voltage equivalent to 97 V.

The overall performance achieved by the currently designed converter is a level higher than the other discussed devices. The results and values shown in the figures reflect the effectiveness of the device. The value of current and THD is around the sustainable range.

### Discussion of simulation result with inductive load

**Figure 9:**
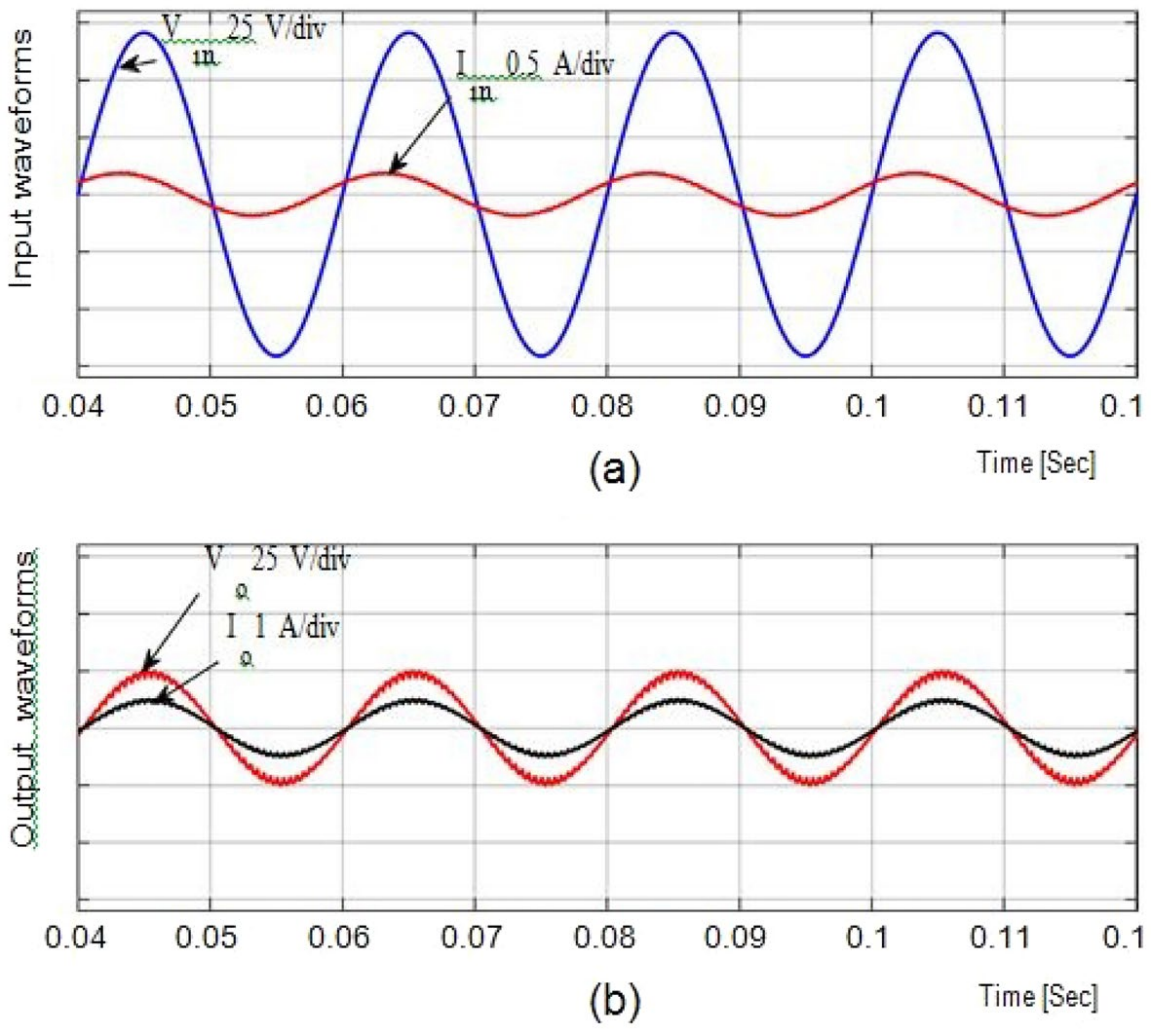
Results of input voltage and output current of Resistive load.

**Figure 10:**
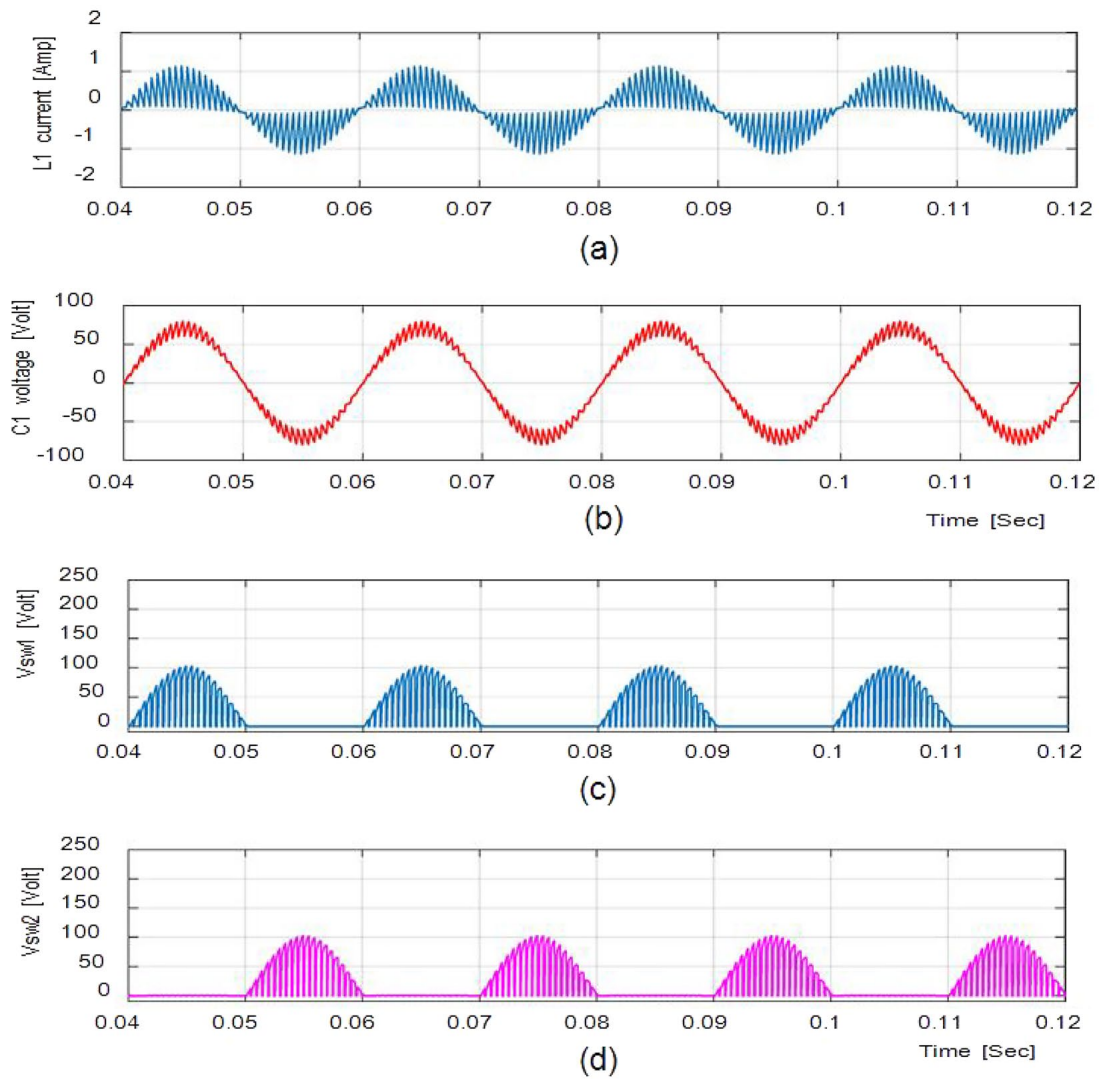
Output of the converter at D = 0.5

**Figure 11:**
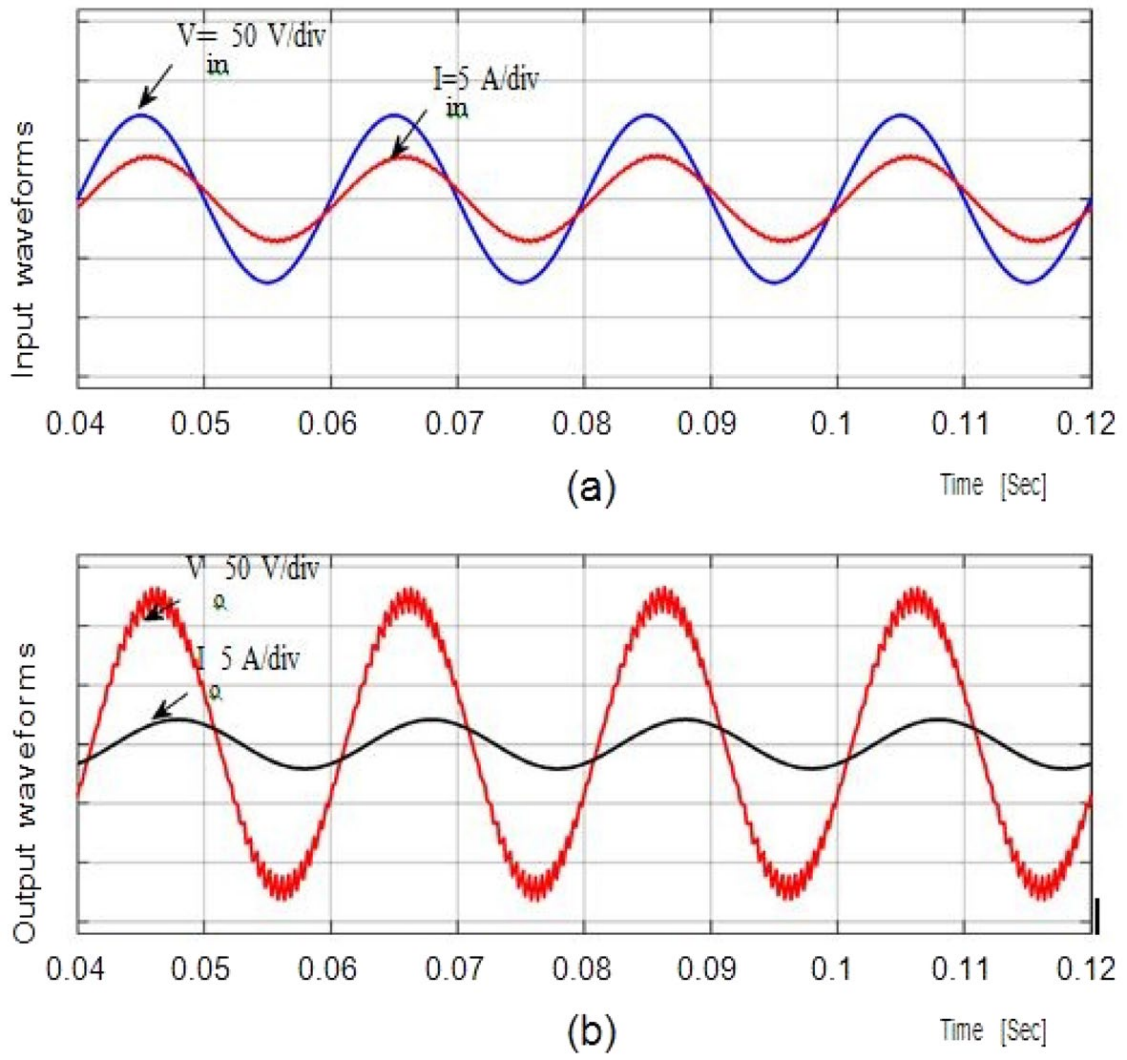
Output waveforms for an inductive load.

In Figs. 9, 10, and 11 show the waveforms obtained for Resistive and inductive load (Ro = 100Ω & Lo = 150mH) with a 50 V AC supply.

The waveforms for the step-up mode are shown in Fig. 9a. It represents the currents have virtually pure sine waves with low THD that are semi-continuous. The THD value for both currents is 3.26 percent and 0.43 percent, respectively. The voltage gain for the inductive load for all cases is identical to those for the resistive load, which has a voltage gain of around 1.857. Additionally, Fig. 10 shows that the voltage potential across S1 and S2 as well as the current (iL1), and voltage (vC1), are virtually identical to those with the resistive load.

In bucking mode, the suggested converter's performance with an inductive load is examined. Figure 11 gives the results obtained using simulation studies. Since the potential difference and magnitude of current are almost in phase with each other, the power factor is almost one. To get the required efficient performance with reduced component size and minimum filter circuits, the suggested prototype is further modified for greater operating frequency (fsw = 55 kHz) mentioned in Table 2. It will give highly efficient, high performance, exceeding 97%.

**Table 2:** Ranges of the components at *f*_*sw*_ = 55 kHz.

Sl. no	Components	Range
1	Capacitor (*C*_1_)	2.5 µF
2	Inductor (*L*_1_)	0.55 mH
3	Input inductor (*L*_*in*_)	0.6 mH
4	Output capacitor (*C*_*out*_)	4 µF
5	Input voltage (*V*_*in*_)	100 V_rms_/50 Hz
6	Resistive load (*R*_*o*_)	100 Ω
7	Inductive load (R_o_ and L_o_)	100Ω and 150mH

**Figure 12:**
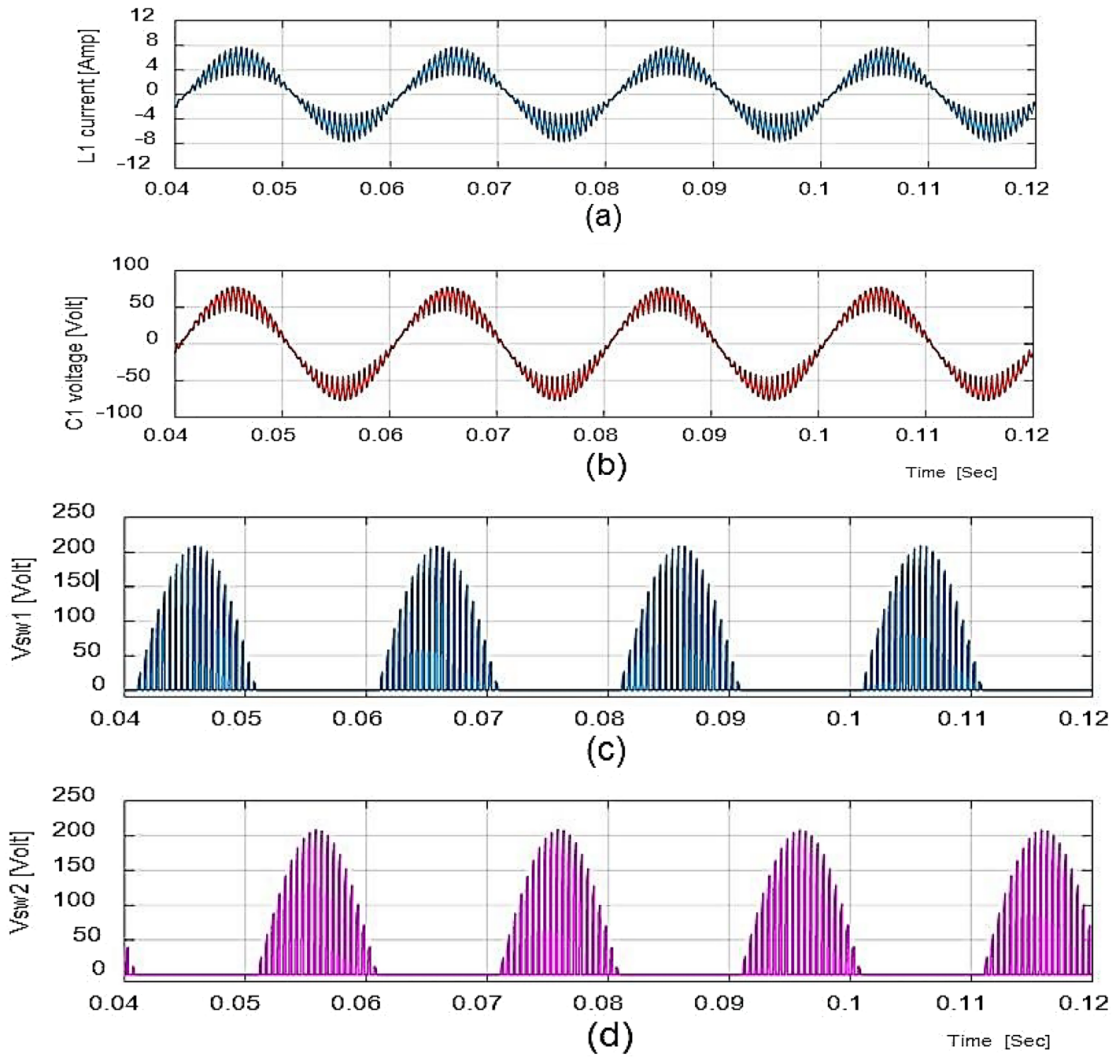
Output waveforms for inductor load at D = 0.65 and f_sw_ = 3 kHz.

**Figure 13:**
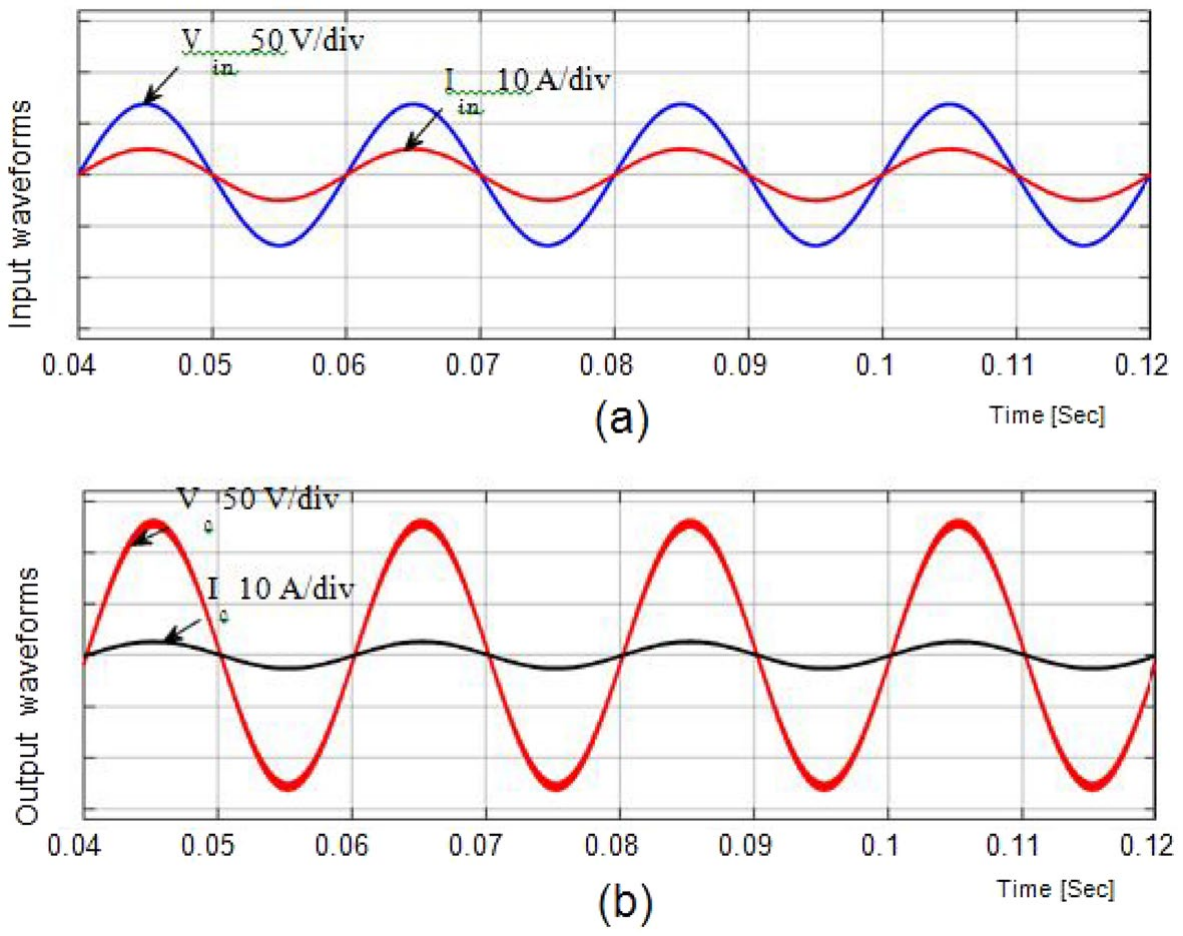
Output current and voltage response for resistance load.

As shown in Table 2, its dimensions will also be quite small, as input and output filters. Figure 12 represents the waveforms for input and output potential difference with the duty ratio D = 0.65 and frequency fsw = 55 kHz. Figure 13 gives the current of the converter.

**Figure 14:**
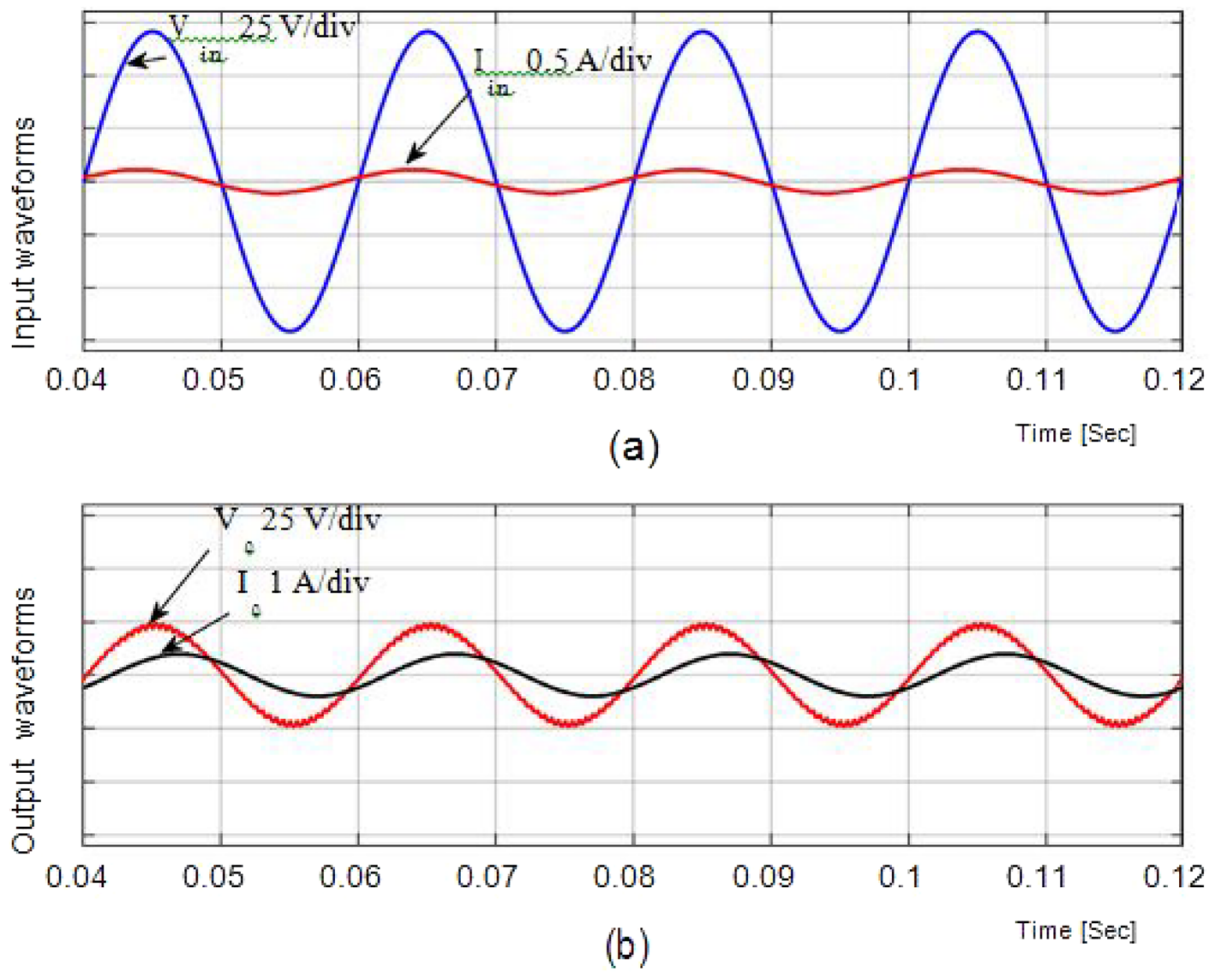
Output waveforms for inductor load.

With the updated components range the device provides an output voltage with a G = 2.13 voltage gain. At D = 0.65, the power factor (P.F.) is 0.97, whereas, converter efficiency is 98%. Figure 14, shows the THD value of the output voltage with low input current.

### Comparative analysis with previously reported converters

**Figure 15:**
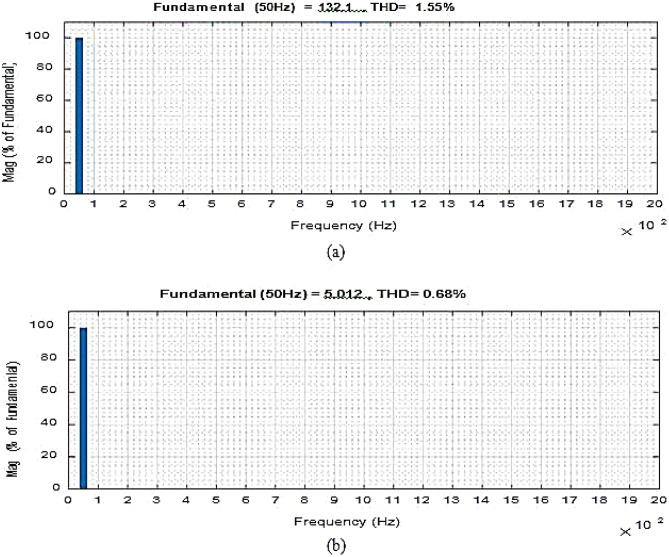
THD values of output voltage and input current at f_sw_ = 55 kHz.

To analyze and compare the results obtained by this study, the values are compared with the previously reported devices as shown in Table 3. The suggested converter is built with fewer switches and passive components than the rival. The size, total power losses, and overall cost of the converter can all be decreased by lowering the power electronics components. Figure 15 defines the THD of the output current.

**Table 3:** Comparison of the proposed study with earlier reported devices.

Description	This study	Reported devices				
Numbers		Reference^25^	Reference^26^	Reference^27^	Reference^28^	Reference^30^
Switch	4	4	4	4	6	6
Diode	–	4	4	6	–	6
Inductor	1	5	2	4	2	1
Bypass capacitor	1	2	2	2	2	2
High frequency						
Control units	1	7	7	8	1	1
Allowed conductors	2	3	4	3	3	4
i/p and o/p waveforms	Continuous	Apparently changing

## Conclusion

In this study, a solitary AC to AC converter with few semiconductor switches, less rating, and few passive components was introduced. As a result, the size and power losses are minimized with effective elevated converter efficiency. The control protocol, which was described, was fairly straightforward. The procedure and analysis of the design and circuit were demonstrated for the parameters. A comparison with earlier converters was done, and the results show that the suggested converter is better than prior reported devices in terms of switches and passive components. The current research was validated through simulation analysis under various scenarios. The input and output values along with THDs fall under admissible range bounds. The circuit design structure might be particularly interesting to enhance the efficiency.

## Data Availability

The datasets used and/or analyzed during the current study are available from the corresponding author upon reasonable request.
