# Author Correction: Design and analysis of solitary AC–AC converter using reduced components for efficient power generation system

**DOI:** 10.1038/s41598-024-61208-x

**Published:** 2024-05-03

**Authors:** K. Nandakumar, V. Mohan, Faisal Alsaif, S. Senthilkumar

**Affiliations:** 1 Department of Electrical and Electronics Engineering, E.G.S. Pillay Engineering College, Nagapattinam, Tamilnadu 611002 India; 2 Department of Electrical Engineering, College of Engineering, King Saud University, 11421 Riyadh, Saudi Arabia; 3 Department of Electronics and Communication Engineering, E.G.S. Pillay Engineering College, Nagapattinam, Tamilnadu 611002 India

Correction to: *Scientific Reports* 10.1038/s41598-024-60230-3, published online 23 April 2024

The original version of this Article contained an error in Affiliation 1, which was incorrectly given as ‘Department of Electrical and Electronics Communication, E.G.S. Pillay Engineering College, Nagapattinam, Tamilnadu, 611002, India’. The correct affiliation is listed below:

Department of Electrical and Electronics Engineering, E.G.S. Pillay Engineering College, Nagapattinam, Tamilnadu, 611002, India

The original Article has been corrected.

